# Reexamination of *Rhopalosiphum* (Hemiptera: Aphididae) using linear discriminant analysis to determine the validity of synonymized species, with some new synonymies and distribution data

**DOI:** 10.3897/BDJ.8.e49102

**Published:** 2020-01-27

**Authors:** Michael Skvarla, Matthew Kramer, Christopher L. Owen, Gary L Miller

**Affiliations:** 1 Penn State, University Park, United States of America Penn State University Park United States of America; 2 Statistics Group, Agricultural Research Service, U.S. Department of Agriculture, Beltsville, MD, United States of America Statistics Group, Agricultural Research Service, U.S. Department of Agriculture Beltsville, MD United States of America; 3 Systematic Entomology Laboratory, Agricultural Research Service, U.S. Department of Agriculture, Beltsville, MD, United States of America Systematic Entomology Laboratory, Agricultural Research Service, U.S. Department of Agriculture Beltsville, MD United States of America

**Keywords:** Aphidoidea, Aphididae, taxonomy, agriculture, species delimitation, linear discriminant analysis, *Melanaphisarundinariae*, *Rhopalosiphumnymphaeae*

## Abstract

Although 17 species of *Rhopalosiphum* (Hemiptera: Aphididae) are currently recognized, 85 taxonomic names have been proposed historically. Some species are morphologically similar, especially alate individuals and most synonymies were proposed in catalogues without evidence. This has led to both confusion and difficulty in making accurate species-level identifications. In an attempt to address these issues, we developed a new approach to resolve synonymies based on linear discriminant analysis (LDA) and suggest that this approach may be useful for other taxonomic groups to reassess previously proposed synonymies. We compared 34 valid and synonymized species using 49 measurements and 20 ratios from 1,030 individual aphids. LDA was repeatedly applied to subsets of the data after removing clearly separated groups found in a previous iteration. We found our characters and technique worked well to distinguish among apterae. However, it separated well only those alatae with some distinctive traits, while those apterate which were morphologically similar were not well separated using LDA. Based on our morphological investigation, we transfer *R.arundinariae* (Tissot, 1933) to *Melanaphis* supported by details of the wing veination and other morphological traits and propose *Melanaphistakahashii* Skvarla and Miller as a replacement name for *M.arundinariae* (Takahashi, 1937); we also synonymize *R.momo* (Shinji, 1922) with *R.nymphaeae* (Linnaeus, 1761). Our analyses confirmed many of the proposed synonymies, which will help to stabilize the nomenclature and species concepts within *Rhopalosiphum*.

## Introduction

The difficulty of aphid taxonomy and identification has been recognized as far back as the 18^th^ Century by Carl Linnaeus (Walsh 1863) and is driven by multiple, often interconnected factors, including morphology and life history traits. Many aphids are heteroecious (Blackman and Eastop 2000) and host-specific morphology can vary enough that taxonomists have described the same species multiple times based on different host-associated life stages (Stern et al. 1997). Furthermore, aphid morphology can change substantially with abiotic factors such as temperature (e.g. Blackman and Spence 1994) and number of daylight hours (e.g. Hille Ris Lambers 1966), and biotic factors such as colony size (e.g. Watt and Dixon 1981), all of which cloud species delimitation. Lastly, most species diagnoses require measurements of body parts. Rarely are there one or more discrete characters that can separate species. Therefore, given the plasticity in aphid morphology and the paucity of discrete characters, aphid taxonomy desperately needs better ways to diagnose species.

Challenges in aphid taxonomy have resulted in different tools and methods to discern species. For example, Foottit et al. (2008) used mitochondrial DNA barcodes to distinguish among aphid species with high success. This high rate of success of DNA barcoding has led to the formation of regional barcoding databases to aid in the identification of aphid species (e.g. Coeur d'Acier et al. 2014). However, DNA barcoding is limited to freshly collected or alcohol-preserved specimens and cannot be used with cleared and slide-mounted specimens, which negates its use when comparing historic specimens.

Both statistical and non-statistical morphological tools have been developed for use with slide-mounted specimens. For example, online interactive keys have been developed to distinguish among large numbers of aphid species, but they lack statistical grounds for identification (e.g., Favret and Miller 2012). Multivariate statistics, specifically linear discriminant analysis (LDA), which requires taxonomic designation *a priori* and can use multivariate analysis of variance (MANOVA) with discrete and continuous characters to test whether the group centroids differ (see Henderson 2006 for multivariate statistics introduction applied to taxonomy), have been applied to aphids to distinguish among aphid species and ecotypes, (e.g. Favret and Voegtlin (2004) for three species of *Cinara*, Lozier et al. (2008) for three species of *Hyalopterus*, Valenzuela et al. (2009) for three species of *Rhopalosiphum* and Bašilova (2010) for two species of *Cryptomyzus*). However, such studies have been limited to just a few species and have not been used to evaluate historic synonymies.

In this paper, we expand upon previous examples using discriminant analysis in aphid taxonomy to test whether multivariate statistics support currently recognized *Rhopalosiphum* species and their synonymies. Specifically, we use LDA to statistically compare 34 valid and synonymized *Rhopalosiphum* species to test the validity of historic synonomies. Valid species and synonymies were tested using iterative LDA analyses in which we removed the most distinct species clusters and reanalyzed the remaining species. We used LDA analyses with only valid species and applied the resulting discriminant functions to synonymized species to determine whether the synonymies are statistically correct using species specific LDA functions. The methods and analyses presented here are unique and repeatable with respect to previous aphid studies. No previous studies have tested taxonomic hypotheses in this manner. We use open source software and describe the analyses with sufficient detail to make them repeatable, which has been lacking from the literature. Lastly, our analyses are statistically robust with respect to LDA model assumptions as we thoroughly describe character transformations and missing data and their relationship to model assumptions.

Our example genus of aphids in need of comprehensive review and revision is *Rhopalosiphum* Koch, 1857 (Aphididae: Aphidinae: Aphidini: Rhopalosiphina) (Koch 1857). *Rhopalosiphum* currently comprises 17 recognized species, including some of the earliest named aphids (i.e. *R.padi* (Linnaeus, 1758) and *R.nymphaeae* (Linnaeus, 1761)) (Fig. 1, Table 1). Most *Rhopalosiphum* species are heteroecious and typically overwinter on rosaceous trees (Rosaceae: *Crataegus* Tourn. *ex* L., *Malus* Mill., *Prunus* L.) and feed on grasses, sedges, and related plants (Poales) throughout the summer (Table 2). A number are important grain (e.g. *R.maidis* (Fitch, 1856) and *R.padi*) and apple (*R.oxyacanthae*) pests that transmit more than 25 aphid-transmitted plant viruses (Chan et al. 1991) and are often intercepted at ports of entry and in aphid-monitoring suction traps (Strażyński 2010, Favret and Miller 2012, Skvarla et al. 2017). Those species that are not economically important have received comparatively little study. For example, *R.nigrum* Richards, 1960 and *R.padiformis* Richards, 1962 have not been discussed in scientific literature outside of their original descriptions and catalog entries. Examples like this complicate the identification of alates collected without host data (e.g., in suction traps) because the majority of taxonomic resources focus on apterae and not alates.

The taxonomic history of *Rhopalosiphum* is complicated. Many taxa with slightly-swollen siphunculi were historically included within *Rhopalosiphum*, but are now placed in a different tribe, Macrosiphini (e.g. *Hyadaphis* Börner, *Lipaphis* Mordvilko, *Rhopalomyzus* Mordvilko), or other aphidine genera (e.g. *Melanaphis* van der Goot, *Schizaphis* Börner) (Richards 1960, Blackman and Eastop 2017, Favret 2019). Börner (1952) was the first to restrict the definition of the genus as it is currently conceived.

Molecular studies (e.g. Kim and Lee 2008) suggest *Rhopalosiphum* is closely related to the rhopalosiphine genera *Melanaphis* and *Schizaphis*. However, no comprehensive molecular or morphological study has tested the monophyly of the genera and species contained therein (Valenzuela et al. 2009). Indeed, Halbert and Voegtlin (1998) suggested that *Rhopalosiphumarundinariae* (Tissot, 1933) is a North American representative of *Melanaphis* based on “wing venation [and] cuticular sculpturing”, though declined to officially move it to *Melanaphis*.

*Rhopalosiphum* species concepts are further complicated by the presence of cryptic species (Bulman et al. 2005, Valenzuela et al. 2009); holocyclic, heteroecious (alternating between sexual and asexual reproduction on primary and secondary hosts) and anholocyclic, autoecious (only asexual reproduction on secondary hosts) forms within the same species (e.g. *R.padi*); and morphological plasticity within species that often makes morphological species determinations, especially of alate specimens, difficult (personal observation).

Finally, while 17 species are currently recognized, at least 85 specific names (which includes misspellings of valid names) have been applied to *Rhopalosiphum* (Favret 2019). Many of the names were synonymized in species catalogues (e.g. Eastop and Hille Ris Lambers 1976, Remaudière and Remaudière 1997), where the authors gave little or no comment on their reasoning for the moves, and the moves have not been confirmed since they were proposed. Thus, there are potentially valid species that have been improperly synonymized.

Herein, we confirm many historic synonymies using LDA, propose two new synonymies, and report new distribution data for *R.rufulum* Richards, 1960, based on material examined for the analyses.

## Materials and Methods

Most specimens examined are housed in the National Museum of Natural History Aphidomorpha Collection (USNM), which is currently housed at the USDA-ARS Beltsville Agricultural Research Center in Beltsville, Maryland, U.S.A. Additionally, types and other material were borrowed from the Essig Museum of Entomology (EMEC), Berkeley, California, U.S.A.; the Florida State Collection of Arthropods (FSCA), Gainesville, Florida, U.S.A.; the Illinois Natural History Survey Insect Collection (INHS), Champaign, Illinois, U.S.A.; the North Carolina State University Insect Museum (NCSU), Raleigh, North Carolina, U.S.A.; The Ohio State University Triplehorn Insect Collection (OSUC), Columbus, Ohio, U.S.A; the personal collection of Andrew Jensen (AJ), Lakeview, OR, U.S.A.; The Canadian National Collection of Insects, Arachinds, and Nematodes (CNC), Ottawa, Ontario, Canada; the Natural History Museum (NHMUK), London, United Kingdom; and the Aphidological Collection of the University of León (CZULE). For a list of types, species, and number of specimens examined, see Table 1. Museum abbreviations follow Evenhuis (2017).

Slides were labelled with individual, sequential numbers (MS 0001–0980) and specimens assigned a number that was appended to the slide number (e.g. MS 0001-1 for the first specimen on the first slide). Specimens were examined using a Zeiss Axio Imager M1 stereomicroscope; micrographs were taken and measurements of various morphological features of adult female apterous and alate specimens made using AxioVision 4.9.1 software (Carl Zeiss AG, Oberkochen, Germany) (Fig. 2, Table 3). Measurements were hand-written in a notebook, then later manually entered into an Excel spreadsheet.

Morphological terms and structures were adapted from Foottit and Richards (1993). Throughout the text, the term aptera (pl. apterae) refers to wingless adult vivipara (pl. viviparae) and alata (pl. alatae) refers to winged adult vivipara (pl. viviparae). Body measurements were adapted from Foottit et al. (2010). Wing measurements (Fig. 2) were adapted from Favret (2009). All measurements are in micrometers (μm) and were taken from the right side of the body when possible. Species names and statuses follow Favret (2019). Measurement data are available as supplementary files (Suppl. materials 1, 2, 3, 4).

### Statistical analyses

We used linear discriminant analysis (LDA) in R (R Core Team 2017) using the MASS package (Venables and Ripley 2002) to test the synonymized and valid species names designations. The code we used to analyze the apterae datasets is available in Suppl. material 5

The analysis proceeded in several steps, which were similar for apterae and alatae, the first of which was data cleaning, described below. LDA is one of the most commonly employed discriminant function analyses, used both to identify useful characters distinguishing specimens of valid species and groups of species, and to form discriminant functions that could then be applied to specimens of uncertain taxonomic status to determine if they cluster with valid species or cluster separately, which would suggest a new species. This LDA approach was applied in a systematic iterative manner; the initial linear discriminant functions separated the most distinctive species, leaving a large amorphous cluster of the other known species. In the next step, the distinctive valid species were removed in that iteration, and the method applied to the remaining valid species. This could be followed by another iteration, until all valid species were separated to the extent possible using available morphological characters.

**Figure 3.**
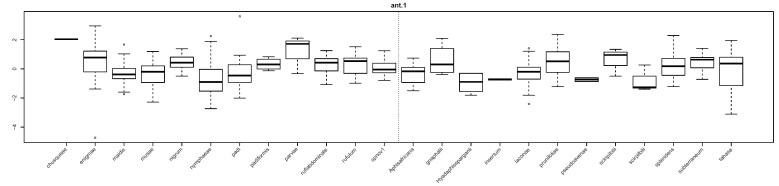
Example of box plots created for a character (length of antennal segment I here) that were used to identify outlier measurements that should be doublechecked before further analyses. Note the outliers for *R.enigmae* and *R.padi*, which were the result of miskeyed data.

Prior to the analyses, the dataset was checked for incorrectly entered data. This was performed for both the valid and synonymized by constructing boxplots for each trait, broken down by species (e.g. Fig. 3). This allowed us to identify outlier measurements in the Excel database, which were compared against the hand-recorded measurements and in most cases were the result of inaccurately entered data (e.g. 10002 instead of 100.02 or 278 instead of 728). Incorrectly entered data that were discovered in this manner were corrected before additional analyses. The boxplots also gave a general picture of which species were more variable for which traits.

#### ‘Size’: as a trait and as an adjustment

We standardized the size measurements of each specimen, based on a ‘size’ variable (but also kept ‘size’ as a variable in the LDA, explained below). There are several reasons for this. One is that some species are more variable in size than others. If measures are not size adjusted, the LDA does not work as well; in fact, we implemented the size adjustments because size affected the usefulness of many of the measures, especially for taxa with a lot of size variability. Second, if one doesn't adjust for size, many of the measures are correlated through size, so less useful. Third, we wanted measures which made sense morphometrically, and these are often 'relative' measures, (e.g. antenna are relatively long for species A (in relation to its size) versus species B). If not adjusted for size, we would have to use ratios for more variables. Standardizing on size was very helpful for many traits and might be useful when developing criteria to separate species in other groups. Given that it is desirable to adjust for size, how does one best estimate 'size'? There is not a one-size-fits-all solution for this and, while we found one that seems to work well for these taxa, it may be improved upon or altered for other taxa. This variable was constructed by combining the body length, head width, and femur length using principal components (PC); head width and femur length were chosen as, among all measured characters, these were most highly correlated with body length. This is a dimension reduction technique, the idea being to create a single variable (the first PC) that best captures the variation in these three correlated measures. In cases where one or two of these measures were missing, the derived principal component measure (henceforth labelled ‘relative size’) for the specimen was imputed using linear regression based on the rest of the data set. Another method employed for developing a size measure is the use the geometric mean of the characters. We calculated the geometric means for data where we had all three characters and found the correlation between the geometric mean and first PC to be 0.9963; for this data set the two methods would yield essentially identical results.

Our relative size measure was retained as a character in the LDA analysis. It was also used to adjust all other size measures using linear regression, i.e. adjusted measures were residuals from regressing each of the size measures (dependent variable) on the relative size (independent variable). This resulted in smaller and larger individuals of the same species having similar adjusted measurements. Non-size measures (such as wing angles or ratios) were not adjusted using relative size, instead they were transformed by taking logs; this created measures that were closer to being normally distributed. All measures were then individually standardized (using all samples) to mean zero, standard deviation one (this helps one to interpret the coefficients of the linear discriminant analysis results). Missing values were then imputed by randomly sampling from the corresponding adjusted measure of other individuals of that same species. In the unusual case where this could not be done (e.g. all specimens were incomplete for this trait), the trait value was set to zero (which is the overall mean for each measure), removing its influence on the calculated discriminant functions. If all specimens of a species were naturally lacking a trait, a new character column was created for that trait, with either 0 (not missing) or 1 (missing). The end result was that a naturally missing trait could be used as a character when creating the linear discriminant function and that incomplete or aberrant individuals were not dropped from the analysis because of missing data.

#### Linear discriminant analyses

A first LDA was performed on the cleaned dataset for only the valid species (this included the three variables used to create relative size, as well as the adjusted size measure) using 49 measured values and 20 ratios calculated from those values. The first three latent axes were sufficient to explain 80% or more of the variability. We looked at which variables loaded most heavily on each axis and compared that to the boxplots created at an earlier step as a check that the methodology was working as expected. The linear discriminant functions derived from the valid species were then applied to all specimens (valid and synonymized) so the specimens could be mapped to the two dimensional space created from pairs of the latent axes. Species were suitably coded so they could be distinguished on the plots. Since there was only one individual of *R.sanguinarium* Baker, 1934 for both apterae and alatae, it was analyzed with the synonymized, rather than the valid, species.

We then identified clusters of specimens comprising a valid species. Sometimes a valid species was well separated from other valid species and sometimes not. For those well-separated valid species, we looked to see if any of the synonymized species occupied the same space and manually outlined the group. When this happened, we concluded that the synonymized species and valid species were likely the same and we removed them from further analyses. If a synonymized species formed a distinct cluster, we concluded that it was not synonymized with any of the valid (or other synonymized) species and considered it a separate species and also removed it from further analyses.

With the remaining species, we repeated the previously described procedure, producing another LDA using only the valid species that did not separate well in the previous analysis, and using the same independent variables. With fewer species and the same number of independent variables, the software sometimes had difficulty due to insufficient degrees of freedom. When this occurred, we reduced the number of independent variables by removing those that were highly correlated with others in a stepwise manner until there were no independent variables that were highly correlated with other independent variables, and then produced an LDA with the reduced set of independent variables. For alatae, this methodology needed to be repeated a third time to finish separating all the known species.

### Notes on *Rhopalosiphum* species not included in linear discriminant analyses

*Rhopalosiphumdryopterae* Kan, 1986. Halbert and Voegtlin (1998) and Jenson and Holman (2000) suggested *R.dryopterae* is a species of *Dysaphis*. The authors have been unable to locate the type specimens or a copy of the original description by Kan (1986), though Holman, now deceased, based his suggestion on it (Jensen, pers. comm). We hesitate to move *R.dryopterae* without having seen specimens or the description, although concede that it is likely not a species of *Rhopalosiphum* given the weight of expert opinion and do not treat it further herein.

*Rhopalosiphumesculentum* Raychaudhuri and Roychoudhuri, 1978. Remaudière and Remaudière (1997) suggested *R.esculentum* may be a synonym of *Aphiscraccivora* Koch, 1854. Unfortunately, the description of *R.esculentum* does not include characters that would definitively place the species in either Aphidina or Rhopalosiphina (i.e. lateral tubercles I and VII dorsal to adjacent spiracles). Raychaudhuri and Roychoudhuri (1978) report the presence of “rhopalosiphine reticulations” on the thorax and abdomen. However, *A.craccivora* also has reticulations and the lack of accompanying illustrations in the description of *R.esculentum* leaves it ambiguous as to whether the reticulations are composed of solid lines as in *A.craccivora* (similar to Fig. 4a, b) or spicules as in *Rhopalosiphum* (Fig. 4c). Certain characters in the description agree with *A.craccivora* (e.g. segments I, II, apex of V, and VI dark or dusky, the shape of and imbrications on the siphunculi). Finally, *A craccivora* is the only aphid species reported to feed on *Manihotesculenta* Crantz, the reported host plant (Blackman and Eastop 2017), which is native to South America and grown as a food crop in India, where *R.esculentum* was described. We, therefore, agree that *R.esculentum* is likely to be a synonym of *A.craccivora* and do not include it in subsequent analyses. However, as we have not examined the holotype of *R.esculentum* to confirm our suspicions, we decline to formally synonymize the two species herein.

*Rhopalosiphummomo* Shinji, 1922. Halbert and Voegtlin (1998) reported that the description of *Rhopaloiphummomo* “is suggestive of” *R.nymphaeae*, but did not formally synonymize the two species. An undated, unattributed translation of the original description is available in the USDA-ARS Systematic Entomology Laboratory library and is reproduced here in full:

(40) *Rhopalosiphummomo* SHINJI, n. sp. / pp. 791

Characteristics: Body green or pale. Antennae somewhat longer than body, III shorter than IV and V taken together, with 16–18 subcircular sensorial, IV and V subequal in length, the former with one sensorium in the middle and the latter with a subapical one; flagellum of VI about 3 times as long as the base. Antennae as a whole infuscated and each segment has a few hairs. Cornicles with a basal 2/3 part green or pale, the remaining 1/3 part swollen and infuscated.

Host plant: *Prumus* [sic] *persica* L.

Date of collection: 2 June 1920

Locality: Miyakonojo, Nakajo (Nagano Pref.)

*Rhopalosiphumarundinariae* (Tissot, 1933). Halbert and Voegtlin (1998) suggested that *R.arundinariae* belongs within *Melanaphis* based on “wing venation [and] cuticular sculpturing” (they also suggested bamboo as a host plant is a *Melanaphis* but not a *Rhopalosiphum* characteristic; however, *R.chusqueae* Pérez Hidalgo and Villalobos Muller, 2012, which feeds on bamboo, was subsequently described and placed unambiguously within *Rhopalosiphum* based on morphological and molecular characters, thus establishing bamboo-feeding as a *Rhopalosiphum* characteristic). We agree with Halbert and Voegtlin (1998) and did not include it in the linear discriminant analyses (see Results for additional details).

We were unable to locate apterae of *R.cerasifoliae* (Fitch, 1855) to include in the analyses. However, we included specimens of the junior synonym *R.tahasa* Hottes, 1950 in order to understand what might happen when synonymized species do not have a presumed conspecific valid species for comparison.

## Results

In total, 1,030 *Rhopalosiphum* specimens (625 apterae, 405 alatae), representing 34 valid and synonymized species, were measured (Table 1). This resulted in 50,470 independent measurements and 20,600 ratios calculated from those measurements (Suppl. materials 1, 2, 3, 4). Additionally, 6 wing vein angles were measured for 62 *Melanaphis* (6 species) and 99 *Schizaphis* (7 species) specimens (Table 4).

### New synonymies and distribution data

The type material of *R.momo* is apparently lost, so, left with only the description, we concur with Halbert and Voegtlin (1998) that *R.momo* is synonymous with *R.nymphaeae* and formally synonymize them for the following reasons:

1) *Rhopalosiphumnymphaeae* is the only species in the genus with siphunculi that are green basally and dark and expanded apically, as was described in *R.momo*.

2) Seven species of *Rhopalosiphum* utilize *Prunus* as a primary host (Table 2), five of which occur in Japan: *R.maidis*, *R.nymphaeae*, *R.oxyacanthae*, *R.padi*, and *R.rufiabdominale*. Of these candidate species, only *R.nymphaeae* is not eliminated based on differences in color.

a) The body of *R.momo* is described as “green or pale”. *Rhopalosiphumpadi* and *R.rufiabdominale* have a distinctive red patch between the siphunculi and *R.oxyacanthae* have dark green stripes that would probably have been noted in the description if present

b) The antennae of *R.momo* are “whol[ly] infuscated”. Antenna segment III of *R.maidis* is pale, rather than concolorous with the other dark segments, and can be pale or dark in *R.padi*.

3) One potential complication is that Richards (1960), who published the last review of *Rhopalosiphum*, reported that alate *R.nymphaeae* have zero secondary rhinaria on antennal segment IV. However, Heie (1986) reports a range of 0–8 secondary rhinaria on antennal segment IV and multiple examples in the USNM collection have 1–4 (although most have zero). The single secondary rhinaria on antennal segment IV reported for *R.momo*, therefore, does not exclude the possibility they are synonymous with *R.nymphaeae*.

The morphological characters suggested by Halbert and Voegtlin (1998) support the placement of *R.arundinariae* (Tissot, 1933) within *Melanaphis*. The reticulate pattern on the dorsal abdomen of *R.arundinariae* (Tissot, 1933) is formed by smooth lines, as in *M.pyraria* (Passerini, 1861), rather than small spicules, as in *Rhopalosiphum* (Fig. 4). The gestalt of the wing veins is more difficult to classify as there can be high levels of plasticity in the veins. However, the angle A3 is consistently different and non-overlapping between *Rhopalosiphum*/*Schizaphis* and *Melanaphis*: *Rhopalosiphum* (28.8–44.9°), *Schizaphis* (29.5–44.6°) and *Melanaphis* (44.9–60°) (Table 4). The angle A3 of *R.arundinariae* is 45.3–55.7°, so supports its placement within *Melanaphis*. We, therefore, transferred *Rhopalosiphumarundinariae* (Tissot, 1933) to *Melanaphis* and did not include it in additional analyses.

Pursuant to Article 57 of the International Code of Zoological Nomenclature (International Commission on Zoological Nomenclature 2000), *Melanaphisarundinariae* (Takahashi 1937) is considered a junior homonym of *Melanaphisarundinariae* (Tissot 1933a). We therefore suggest *Melanaphistakahashii* Skvarla, Kramer, Owen, and Miller, in honor of Ryoichi Takahashi, who originally described the species, as a replacement name for *Melanaphisarundinariae* (Takahashi, 1937).

While sorting undetermined *Rhopalosiphum* specimens in the USNM collection, three slides of specimens collected from *Acorus* in North America were discovered. The specimens match the measurements and brief descriptions of European *R.rufulum* apterous viviperae (Stroyan 1972, Heie 1986). These specimens represent the first collections of *R.rufulum* from secondary hosts in North America and extend the known range southeast of previous collections. In Europe, colonies on *A.calamus* are reported to grow so large that “the plants look bespattered with black mud” (Heie 1986), so, while the species is rarely collected in North America, it is presumably common where the appropriate host plants are present. The collection data are as follows:

CANADA: 3 female apterae, locality unknown (label states “at HO-17214”), ex *Acoruscalamus*, 29-IX-1952, J. Adams, USNM; UNITED STATES: Massachusetts: 2 female apterae, Hampshire Co., Amherst, ex. *Acorus*, 31-V-1964, M. Smith, USNM; New York: 3 nymphs, Suffolk Co., Greenport, ex. *Acoruscalamus*, 25-VI-1963, R. Latham, USNM.

### Morphometric analyses

**Figure 5.**
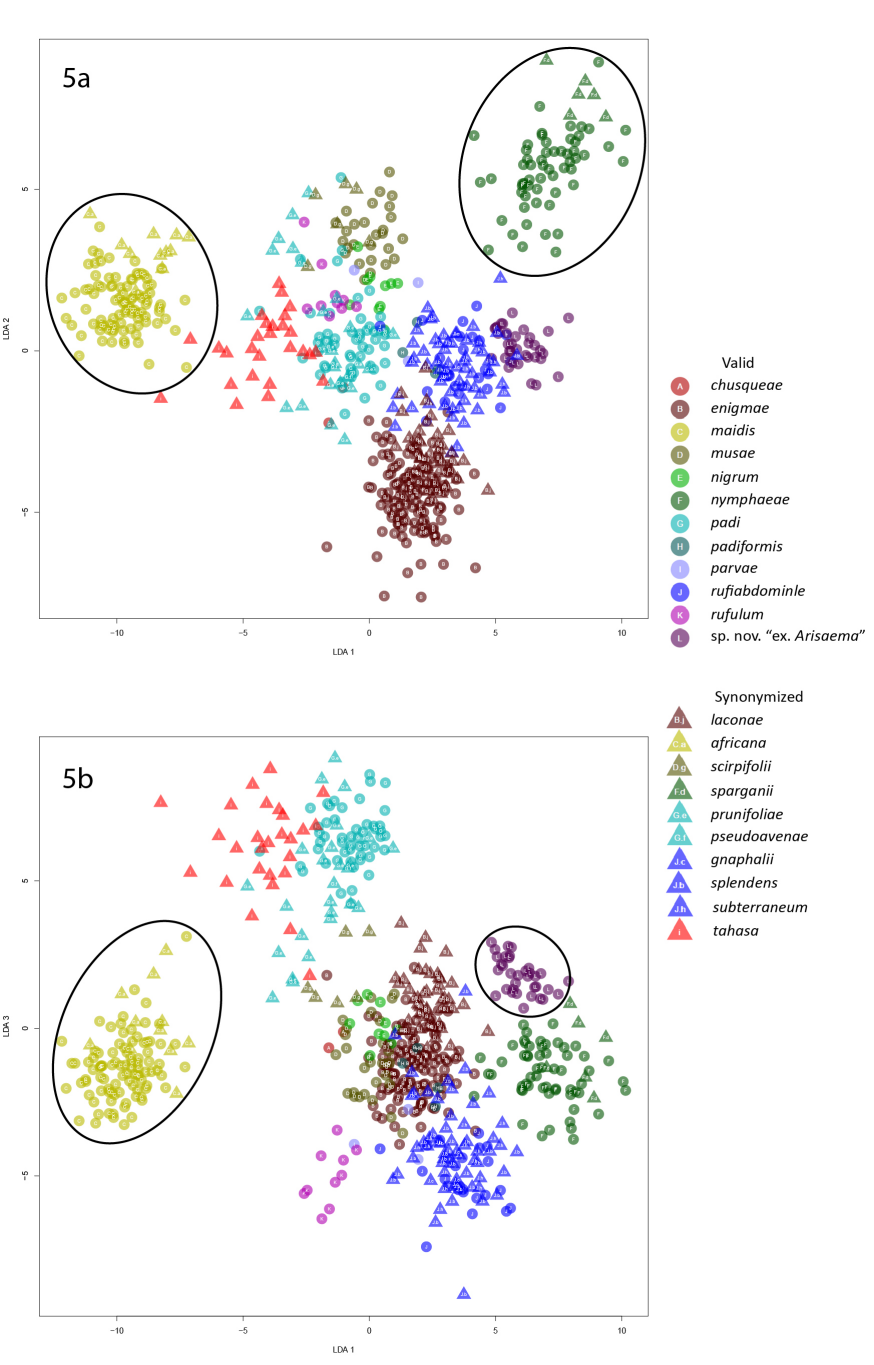
Graphs of the first linear discriminant analysis of apterae.

In the first LDA of apterae, *R.maidis* and its synonom *R.africana* (Theobald, 1914) formed a distinct cluster in the plots of LD1 x LD2 and LD1 x LD3 (Fig. 5). *Rhopalosiphumnymphaeae* L., 1761) and its synonym *R.sparganii* (Theobald, 1925) formed a distinct cluster in the LD1 x LD2 plot (Fig. 4a) and *R.* sp. nov. “ex. *Arisaema*” formed a distinct cluster in the plot of LD1 x LD3 (Fig. 4b). These five species were removed prior to performing the second LDA.

**Figure 6.**
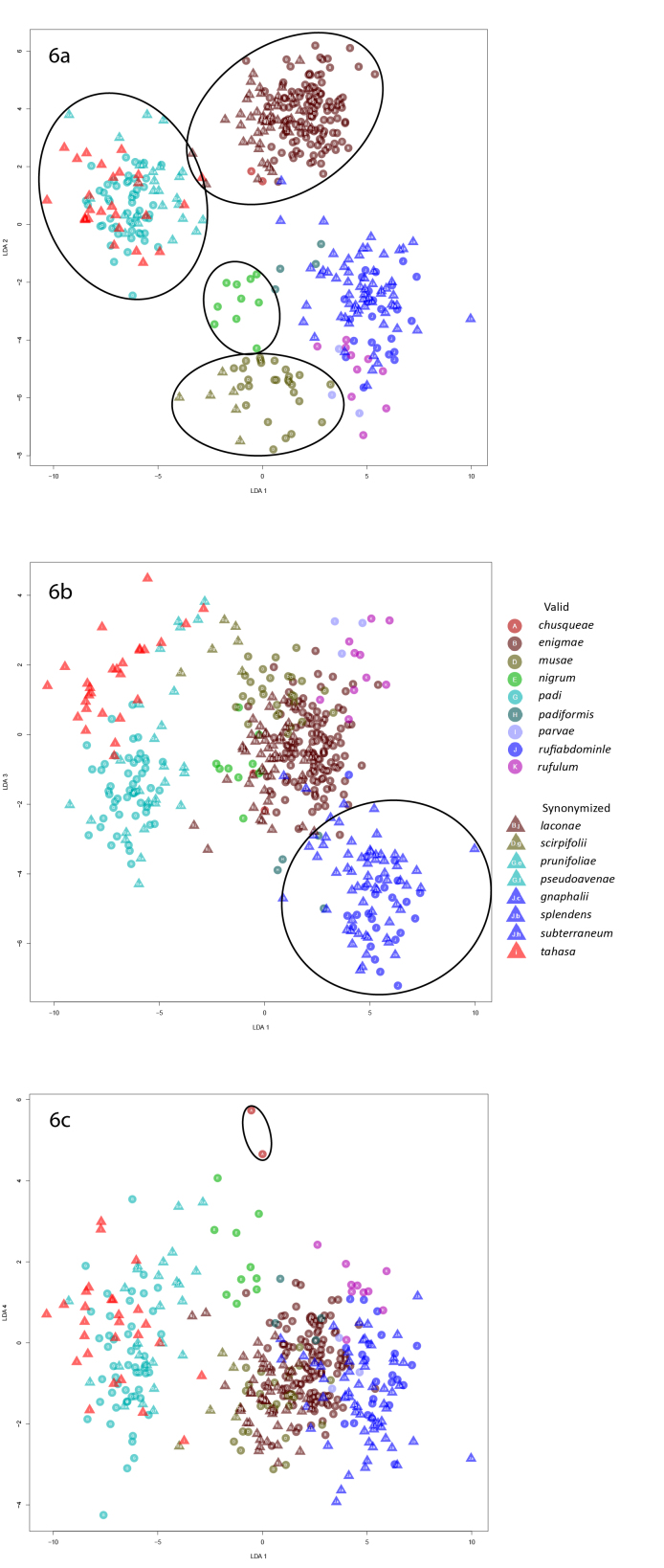
Graphs of the second linear discriminant analysis of apterae.

In the second linear discriminant analysis of apterae, *R.padi* and its synonyms *R.prunifoliae* (Fitch, 1855) and *R.pseudoavenae* (Patch, 1917) formed distinct clusters in both the LD1 x LD2 and LD1 x LD3 plots (Fig. 6); *Rhopalosiphumtahasa* (Hottes, 1950) also clustered with *R.padi*, although this may be an artifact of the fact that the species with which it is currently synonymized, *R.cerasifoliae*, was not included in the analyses. In the LD1 x LD2 plot, *Rhopalosiphumenigmae* and its synonym *R.laconae* (+ *R.chusqueae*), *R.musae* (Schouteden, 1906) and its synonym *R.scirpifolii* Gillette & Palmer, 1932 and *R.nigrum* Richards, 1960 all formed distinct clusters. In the LD1 x LD3 plot, *Rhopalosiphumrufiabdominale* (Sasaki, 1899) and its three synonyms *R.gnaphalii* Tissot, 1933, *R.subterraneum* Mason, 1937 and *R.splendens* (Theobald, 1915) clustered together although there was some overlap of *R.splendens* with *R.enigmae* + *R.laconae*. *Rhopalosiphumchusqueae* formed a distinct cluster in the LD1 x LD4 plot. *Rhopalosiphumparvae* Hottes and Frison, 1931 and *R.rufulum* clustered together in all plots, not forming separate clusters in any plot.

**Figure 7.**
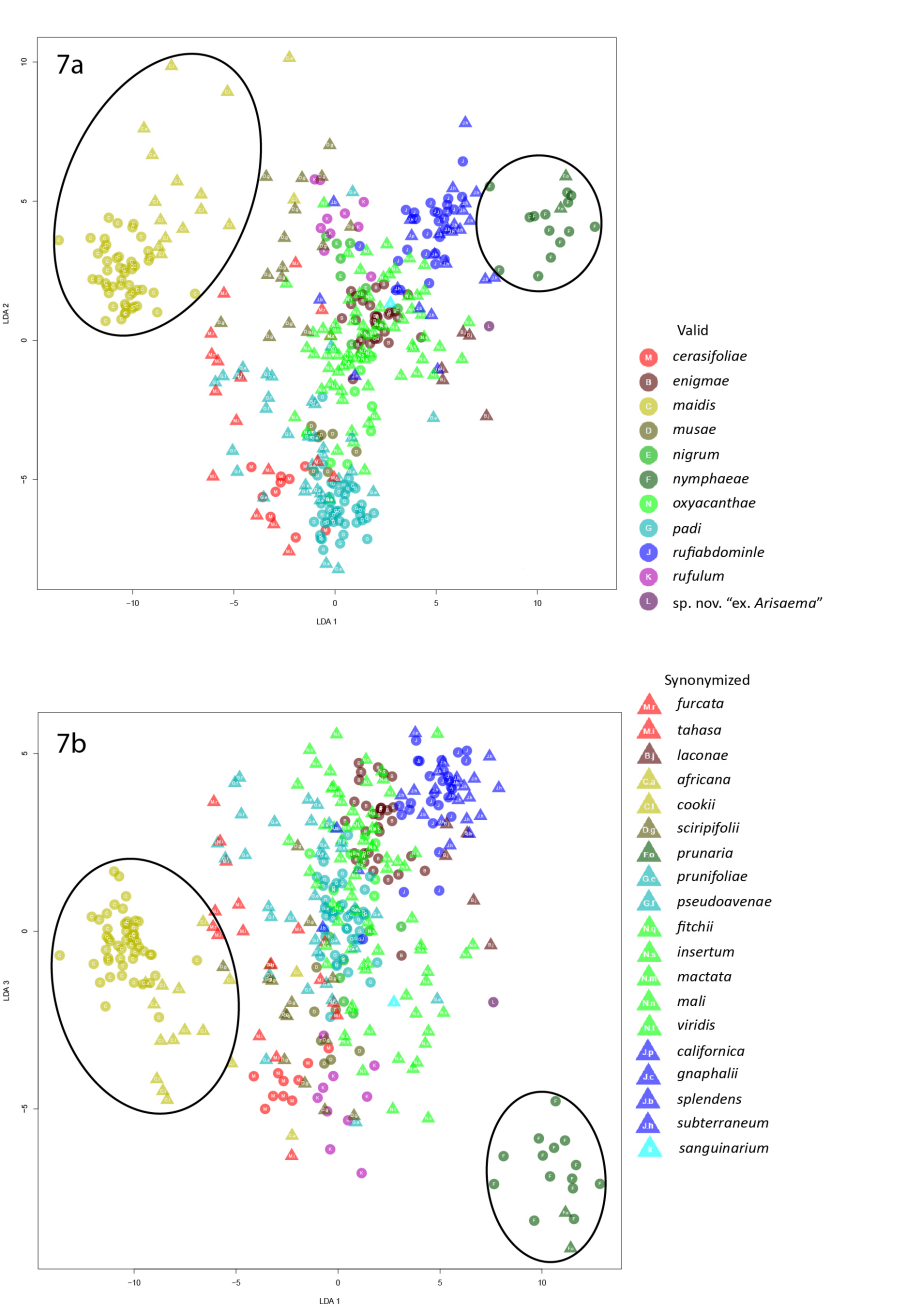
Graphs of the first linear discriminant analysis of alatae.

In the first linear discriminant analysis of alatae, *R.maidis* formed a cluster with its synonyms *R.cookii* (Essig, 1911) and *R.africana* and *R.nymphaeae* clustered with its synonym *R.prunaria* (Walker, 1848) in the plots of LD1 x LD2 and LD1 x LD3 (Fig. 7). These five species were removed prior to performing the second LDA except for the three most outlying *R.africana*, which were paratypes.

**Figure 8.**
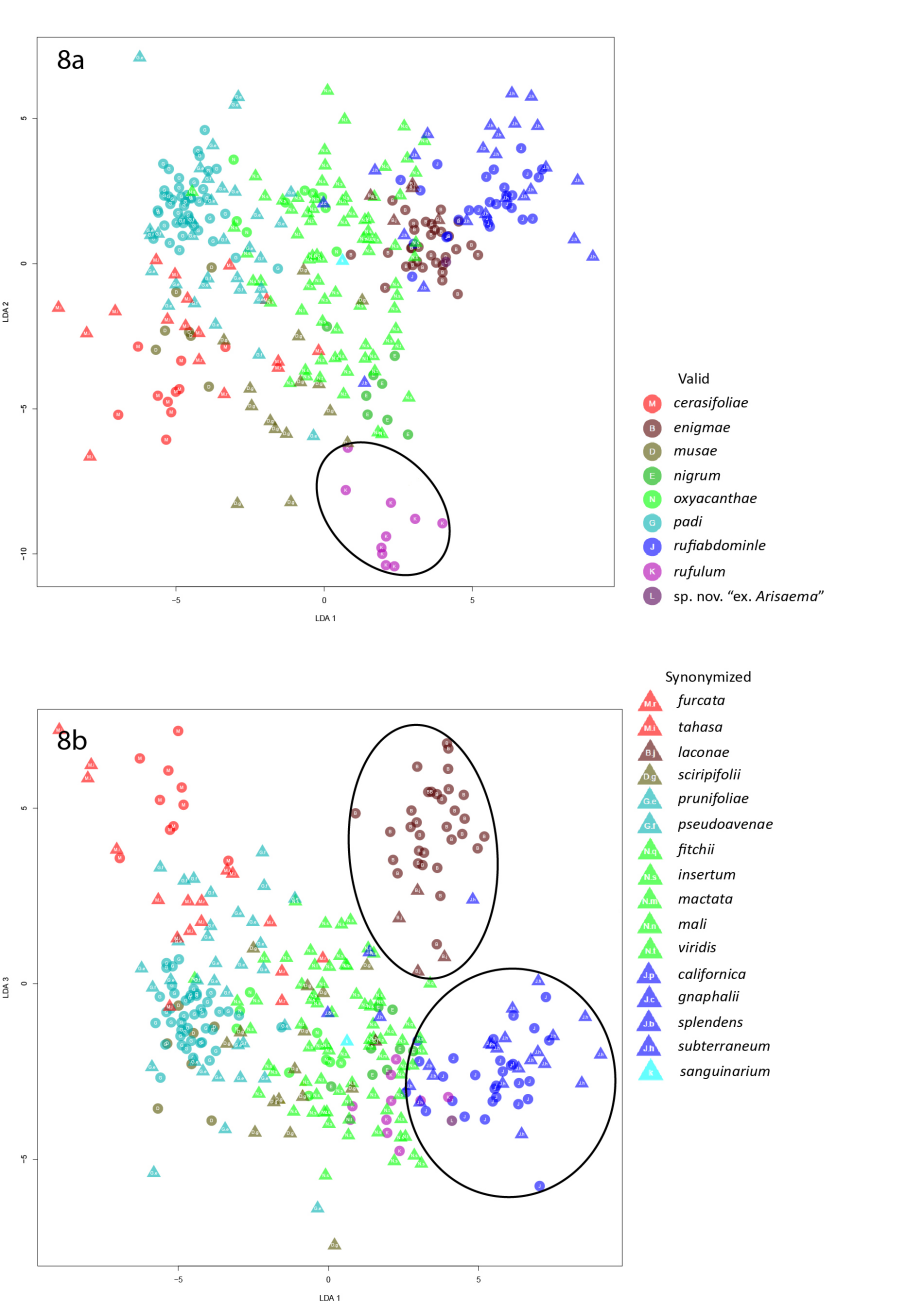
Graphs of the second linear discriminant analysis of alatae.

In the second linear discriminant analysis of alatae, *R.rufulum* (along with one of the outlier *R.africana* not removed from the analyses) formed a distinct cluster in the LD1 x LD2 plot (Fig. 8). *Rhopalosiphumenigmae* and its synonym *R.laconae* formed a distinct cluster in the LD1 x LD3 plot and were removed prior to performing the third LDA. *Rhopalosiphumrufiabdominale* and its synonyms *R.californica* (Essig, 1944), *R.splendens* (Theobald, 1915), and *R.subterraneum* Mason, 1937 formed a distinct cluster in the LD1 x LD3 plot with minimal overlap with *R.oxyacanthae* synonyms and so were also removed prior to the third LDA.

**Figure 9.**
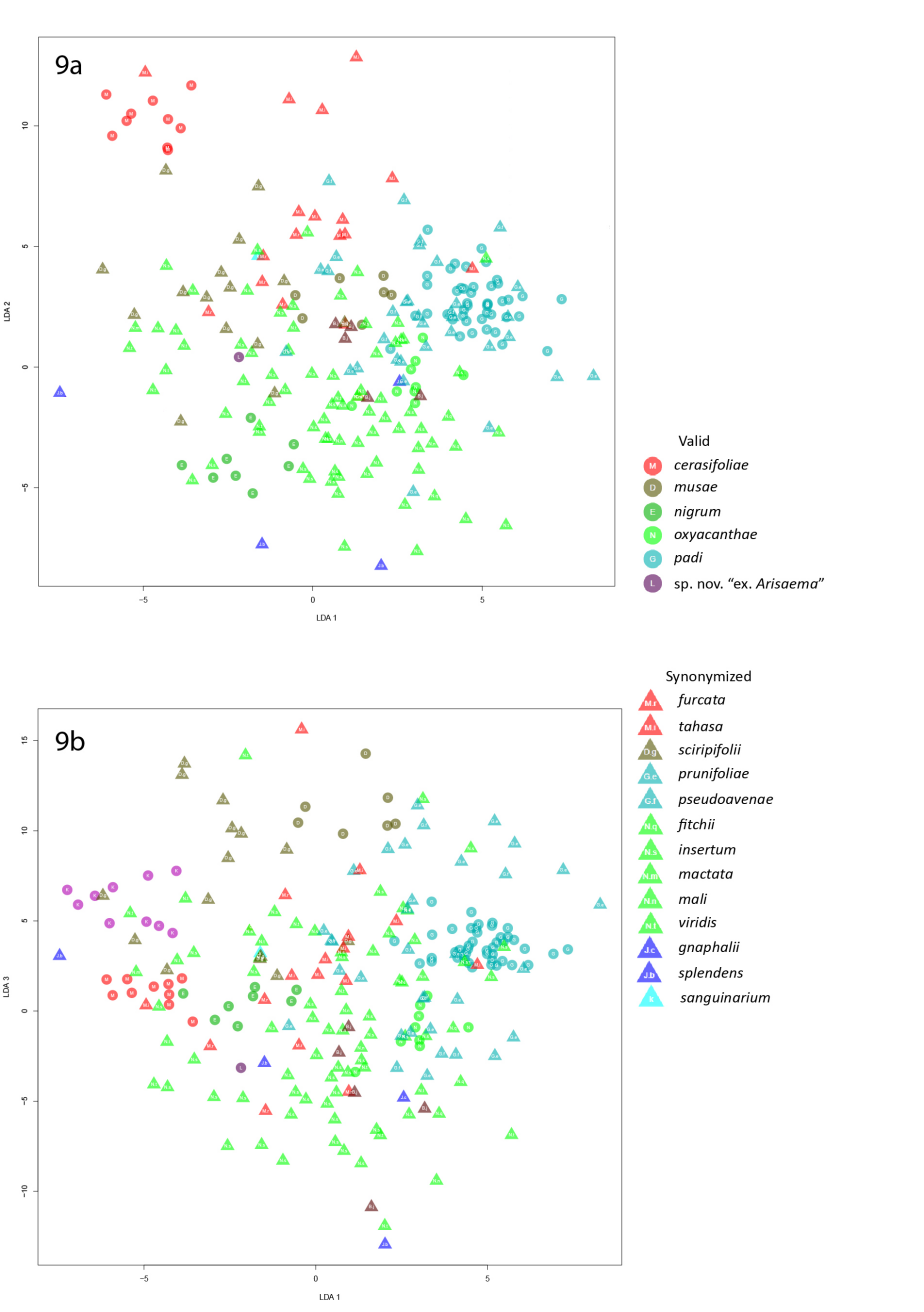
Graphs of the third linear discriminant analysis of alatae.

None of the remaining species formed distinct clusters in the third linear discriminant analysis (Fig. 9).

## Discussion

### Current species and natural history

The synonymization of *R.momo* with *R.nymphaeae* and movement of *M.arundinariae* (Tissot, 1933) to *Melanaphis* brings the total number of described *Rhopalosiphum* species to 17, two of which (*R.dryopterae* and *R.esculentum*) are questionably assigned to the genus, pending examination of the type material, with an additional four undescribed species known.

The discovery of *R.rufulum* on *Acorus* in North America and the presence of an undescribed *Rhopalosiphum* species on *Arisaema* in eastern North America echoes the sentiment of Skvarla et al. (2018) – who found that *R.enigmae*, which was once considered a rare species, was present in every cattail stand examined – that many rarely collected *Rhopalosiphum* species are likely abundant in the correct habitats/secondary hosts and that apparent rarity may be due to inadequate collecting efforts on non-crop plants. Indeed, we expect that additional surveys of semi-aquatic plants, especially monocots, will continue to produce new species of *Rhopalosiphum* in North America and probably East and Southeast Asia.

### Morphological analyses in the molecular era

Determining species boundaries and synonymies using molecular techniques has become the standard by which most modern taxonomy and systematics are measured. Indeed, with the continually lower costs and greater ease with which highly degraded DNA can be extracted and sequenced from historic museum specimens (Gilbert et al. 2007, Bi et al. 2013, Tin et al. 2014, McCormack et al. 2015, Blaimer et al. 2016, Sproul and Maddison 2017), determining species validity and synonomies using old specimens, including type material, has never been easier (e.g. Kirchman et al. 2009, Mutanen et al. 2015, McGuire et al. 2018). However, most aphids and other slide-mounted arthropods present a relatively unique challange in that all of the DNA is destroyed when specimens are cleared for mounting (although the authors have seen a few decades-old aphids that were not cleared before being slide-mounted in balsam and have wondered if it would be possible to free them from the mounting medium and extract DNA). Thus, even in this era when molecular techniques dominate, there is still a need for robust morphological comparisons for certain groups, especially aphids, as has been demonstrated here.

### LDA methodology applied to apterae confirms most species and their synonymies

In general, the linear discriminant analyses were successful when applied to apterae. The analyses successfully grouped all synonymized species with their associated valid species over two iterations and we concluded that most of the synonymizations we tested are sound. This demonstrates that linear discriminant analyses can be used to test synonymizations when DNA is unavailable and provides a new method to examine and use historic, slide-mounted specimens. Additionally, *R.* sp. nov. "ex. *Arisaema*" formed a distinct group when it was included as a valid species. This supports its status as a valid, but undescribed, species and demonstrates that linear discriminant analysis can be used to distinguish potentially undescribed species from valid described species based on morphological similarities.

However, a few notable problems exist. First, *R.parvae* and *R.rufulum* consistently clustered together, but never as a distinct group away from other species. This may indicate that they are synonymous, although several factors indicate they are distinct species: they feed on different secondary hosts (*Carex* and *Acorus*, respectively), have non-overlapping ranges (Illinois versus New England and adjacent Canada), and have several morphological differences that separate them (Table 5). However, this is based on very few individuals (3 and 10 specimens, respectively). Without additional specimens from a wider geographic range and considering the above evidence, we elected to leave them as separate species, although acknowledge the LDA indicated these species concepts should be revisited in the future.

Unfortunately, we were unable to include verified apterous *R.cerasifoliae* in the analyses due to lack of specimens. We included specimens of *R.tahasa*, which is synonymized with *R.cerasifoliae*, in the analyses to see if they would still form a distinct cluster without valid *R.cerasifoliae* to act as a guide in the discriminant function. Instead, the *R.tahasa* specimens clustered well with *R.padi*, which it is not synonymized with, rather than forming a distinct cluster. This association has not previously been suggested in the literature, but without the inclusion of *R.cerasifoliae*, it is impossible to determine if *R.tahasa* should instead be synonymized with *R.padi*. This issue suggests that it is extremely important to include all valid species when creating the discriminant functions, so that synonymized species can be properly plotted. Additionally, assuming that *R.tahasa* is synonymous with *R.cerasifoliae*, it suggests that valid species analyzed with the synonymized species (either because they are incorrectly synonymized with a different species or because, as in this case, a synonymized species is included without specimens of its associated valid species) may not form distinct clusters.

### LDA methodology worked less well for classifying alates using morphological traits

The analyses of alatae were less decisive. Intuitively, species with the most distinctive apterae – *R.maidis*, *R.nymphaeae*, *R.enigmae*, and *R.rufiabdominale* – also had the most distinctive alatae and formed distinct clusters with their associated synonymized species in the first two LDA. However, the remaining species failed to form distinct clusters, even after a third LDA. Additionally, a number of synonymized species – *R.furcata*, *R.fitchii*, *R.insertum*, *R.mactata*, *R.mali*, and *R.viridis* – were only represented by alate specimens and did not cluster with the species with which they are synonymized. Based on our analyses, we cannot confirm that these synonymies are correct, although we also do not propose any be raised as valid species pending additional investigation.

Aphidologists have found that *Rhopalosiphum* alatae captured without host plant data (e.g., in suction traps) are difficult to identify to species due to similar, conserved morphologies. While published keys to alatae are available (i.e. Richards 1960), they can be difficult to use because no key includes all described species and specimens often do not neatly fit within one couplet or another. Rather than revealing new, perhaps subtle, morphological characters that can be used to distinguish alatae, the suboptimal results from the alatae analyses reinforces the perception that they are very similar morphologically and reticent to identification without additional information (e.g. associated apterae, host plant data).

### LDA loadings to create taxonomic keys

The loadings (coefficients) for each character on the discriminant functions could conceivably be used to create taxonomic keys. However, we do not suggest following that path for this group for a number of reasons: measures we used were adjusted by size, based on our data, so this same adjustment would need to be made for all measures (except wing venation angles); variables were then standardized to mean zero, variance one, so each variable would need to be standardized in the same way we did (again, the standardization we used was based on our data); the functions are built largely on continuous characters, rather than naturally dichotomous ones and all characters contribute to each discriminant function. However, since the variables were standardized, their importance (i.e. weighting) to the discriminant function can be evaluated, based on the absolute value of the coefficient. One could determine a cut-off for which variables were important. We looked at this for all the discriminant functions and found that, for any reasonable cut-off, some discriminant functions were largely determined by just a few characters (which would be useful for creating a key), but many were largely determined by at least 12 (which would be less useful). Since placement in the plane for any specimen is determined by two discriminant functions, many with large contributions from many characters, creating a usable key from these results would not be an easy exercise. One possible statistical approach to creating keys from continuous characters that should be explored is the use of regression trees.

## Summary

Two species of *Rhopalosiphum* were moved or synonymized, bringing the total number of species in the genus to 17. While LDA has previously been used to distinguish between a limited number of species or ecotypes, the use of it to confirm previously proposed synonymies using historic slide-mounted specimens that lack DNA is novel and yielded promising results. In particular, the analyses confirmed most synonymizations when apterae were analyzed. However, while the most distinct alate *Rhopalosiphum* and associated synonymies were recovered in the LDA, many species and associated synonymies were not recovered as distinct. The failure of the analyses with some of the alatae using phenotypic traits mirrors problems previously documented for this morphologically similar group.

## Supplementary Material

3E05278F-524A-520C-9C21-78BF43FAF160 10.3897/BDJ.8.e49102.suppl1 Supplementary material 1 Rhopalosiphum measurements, alatae, valid species Data type: Morphological, measurement
Brief description: Length measurements (given in μm) and wing angles for alatae of valid *Rhopalosiphum* species.
File: oo_353663.xlsx https://binary.pensoft.net/file/353663 Michael Skvarla, Gary Miller

CB8FA620-3E8E-568B-862E-11A9B40B73B8 10.3897/BDJ.8.e49102.suppl2 Supplementary material 2 Rhopalosiphum measurements, apterae, synonymized species Data type: Morphological, measurements
Brief description: Length measurements (given in μm) and wing angles for apterae of valid *Rhopalosiphum* species.
File: oo_353665.xlsx https://binary.pensoft.net/file/353665 Michael Skvarla, Gary Miller

CA1E3CEA-FD1A-57FB-807E-05CFC8FBD147 10.3897/BDJ.8.e49102.suppl3 Supplementary material 3 Rhopalosiphum measurements, alatae, synonymized species Data type: Morphological, measurements
Brief description: Length measurements (given in μm) and wing angles for alatae of synonymized *Rhopalosiphum* species.
File: oo_353666.xlsx https://binary.pensoft.net/file/353666 Michael Skvarla, Gary Miller

6EB34862-7E3F-5A81-A6F5-5C2E4DF987B0 10.3897/BDJ.8.e49102.suppl4 Supplementary material 4 Rhopalosiphum measurements, apterae, synonymized species Data type: Morphological, measurements
Brief description: Length measurements (given in μm) and wing angles for apterae of synonymized *Rhopalosiphum* species.
File: oo_353667.xlsx https://binary.pensoft.net/file/353667 Michael Skvarla, Gary Miller

7FA4438E-8A41-54F8-A83B-9DE9EA32DE97 10.3897/BDJ.8.e49102.suppl5 Supplementary material 5 Code to analyze apterae datasets in R Data type: R Code
File: oo_373277.txt https://binary.pensoft.net/file/373277 Kramer, M.

## Figures and Tables

**Figure 1a.**
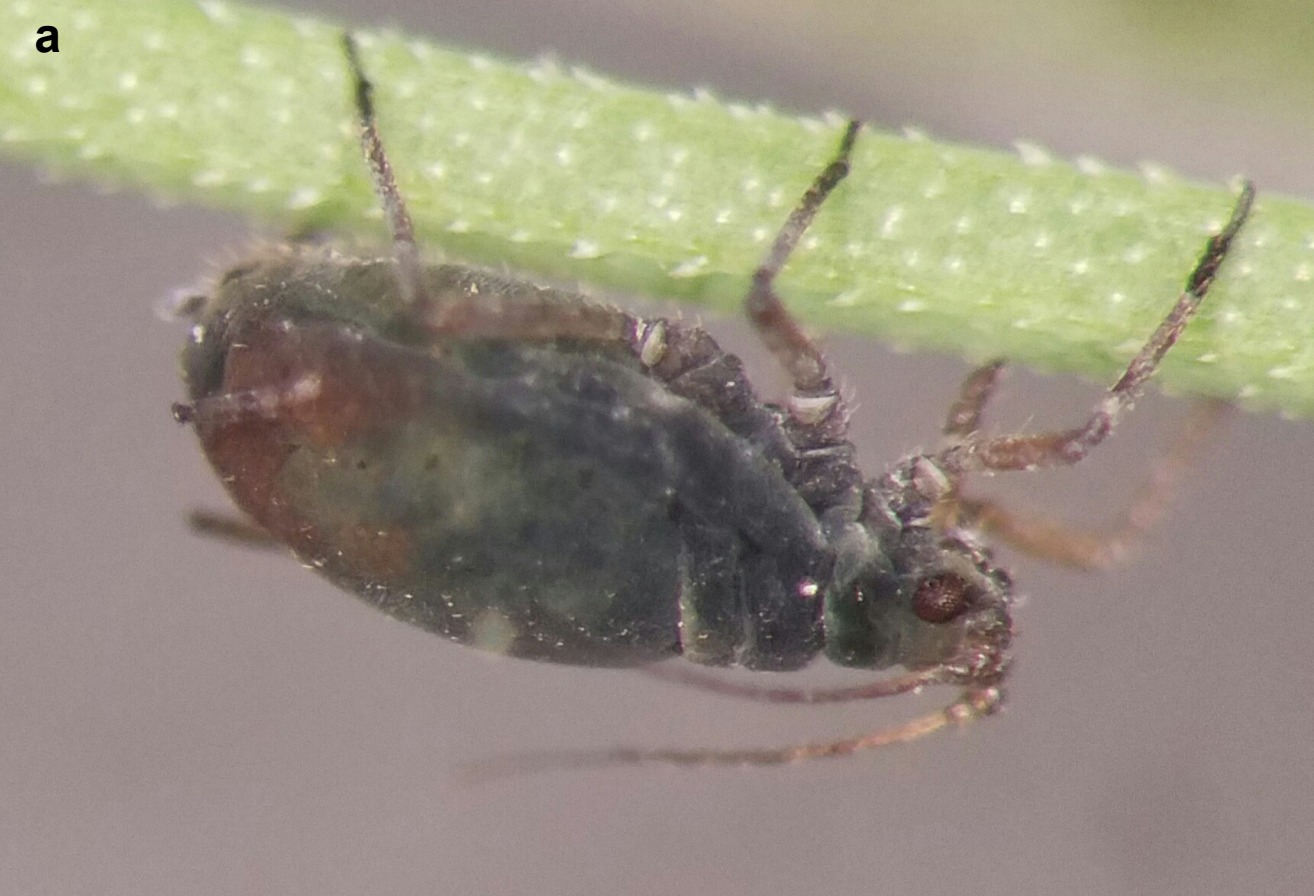
*R.padi*.

**Figure 1b.**
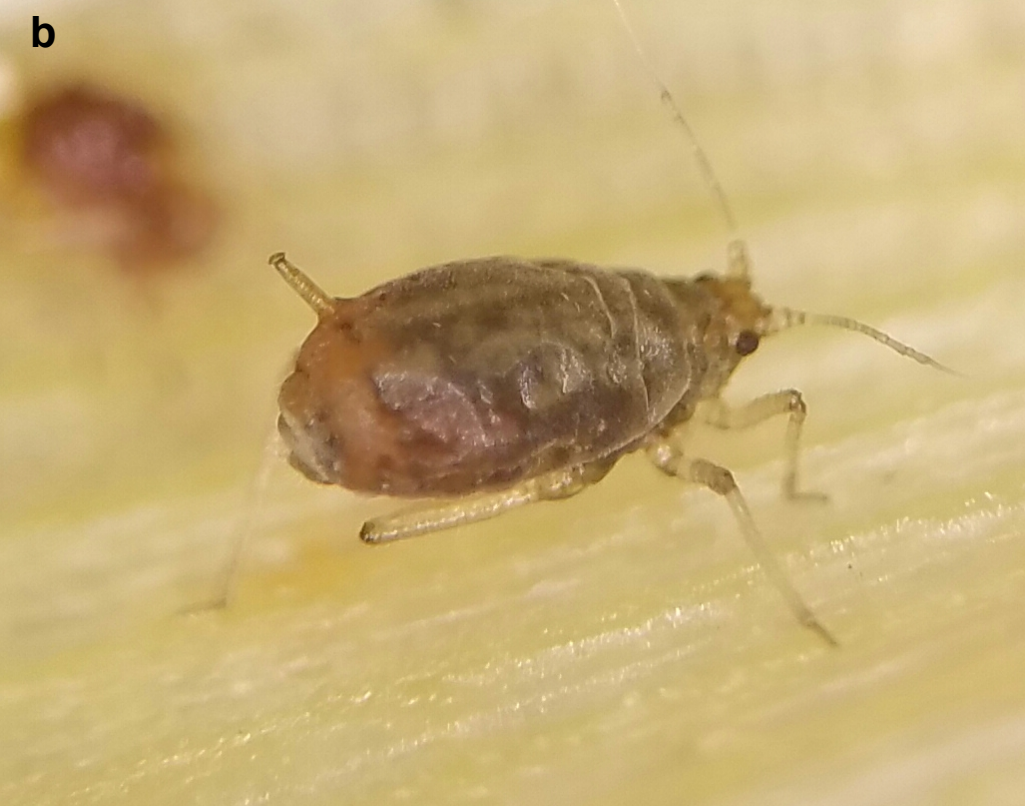
R.enigmae

**Figure 2a.**
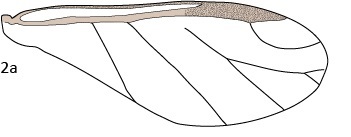
Wing schematic.

**Figure 2b.**
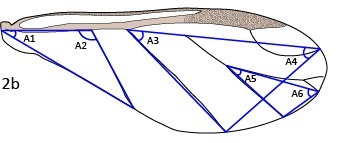
Vein angles.

**Figure 2c.**
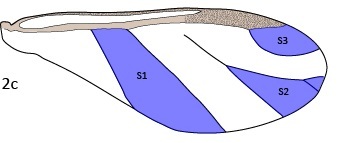
Cell areas.

**Figure 2d.**
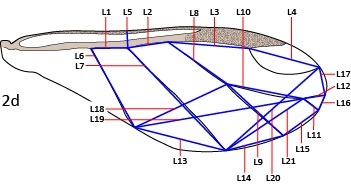
Vein and point-to-point lengths. See Table 3 for explanation of alphanumeric codes.

**Figure 4a.**
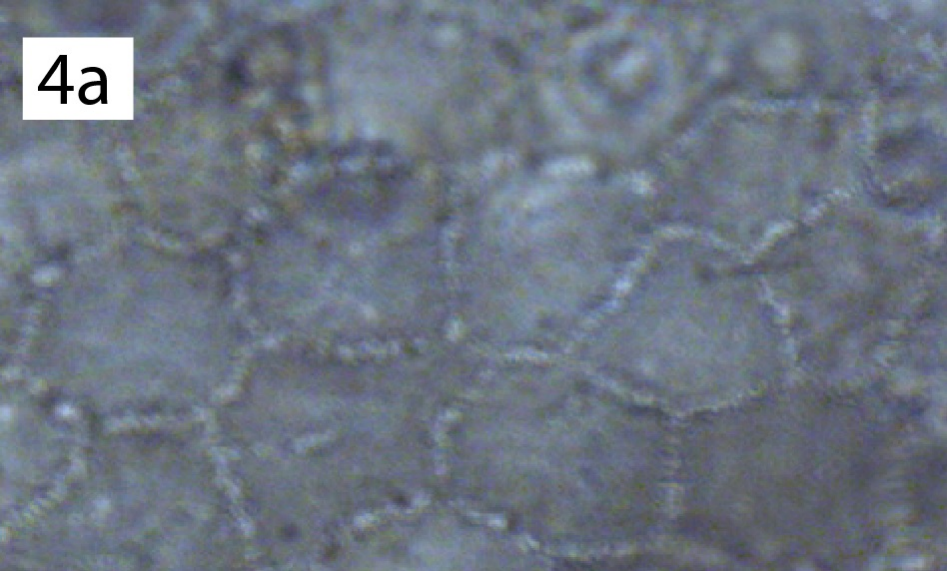
*M.pyraria* (Passerini, 1861).

**Figure 4b.**
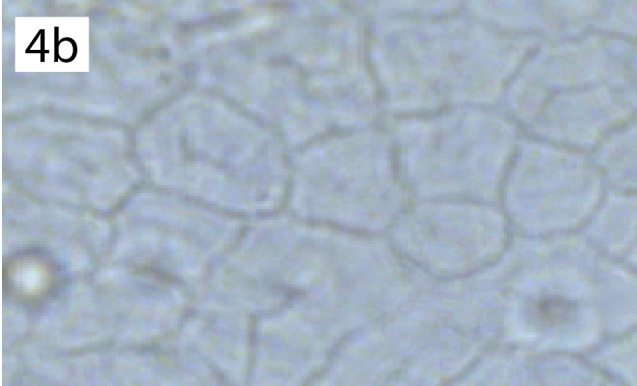
*R.arundinariae* (Tissott, 1933).

**Figure 4c.**
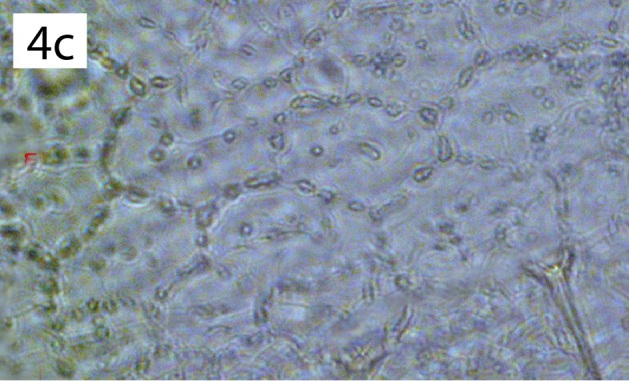
*R.enigmae* Hottes and Frison, 1931.

**Table 1.** *Rhopalosiphum* species, associated original descriptions, type depositories, and number of specimens examined. Bold names are currently recognized as valid species with synonymized species listed immediately after. Citations marked with an asterisk (*) are not included in the literature cited as the authors were unable to locate them.

**Species**	**Original description**	**Primary type depository**	**Secondary type depositories**	**Number of apterae measured**	**Number of alatae measured**	**Notes**
***R.albigubernum***	Zhang and Qiao (1997)			-	-	Unable to locate types.
*R.arundinariae*	Tissot (1933a)	USNM	NHMUK	3	5	Moved to *Melanaphis*.
***R.cerasifoliae***	Fitch (1855)	NHMUK	USNM	0	10	
= *Aphisfurcata*	Patch (1914)	CNC	CNC	0	6	Lectotype and paralectotypes on one slide.
= *Aphistahasa*	Hottes and Frison (1931)	USNM	NHMUK	27	10	
***R.chusqueae***	Pérez-Hidalgo et al. (2012)	CZULE		2	0	
*R.dryopterae*	Kan (1986)			-	-	Unable to locate types.
***R.enigmae***	Hottes and Frison (1931)	INHS	NHMUK, USNM	120	32	
= *R.laconae*	Taber (1993)	USNM	NCSU	54	5	Unable to locate holotype, apparently lost or never deposited. Neotype designated from NCSU paratypes.
*R.esculentum*	Raychaudhuri and Roychoudhuri (1978)			-	-	Unable to locate types; syn. possible de Aphiscraccivora (Remaudiere & Remaudiere 1997)
***R.maidis***	Fitch (1856)	USNM		93	51	
= *Aphisadusta*	Zehntner (1897a)			-	-	Unable to locate types.
= *Aphisafricana*	Theobald (1914)	NHMUK	NHMUK	11	3	
= *Aphiscookii*	Essig (1911)	EMEC		0	13	
= *Stenaphismonticellii*	del Guercio (1914a)			-	-	Unable to locate types.
= *Aphisobnoxia*	Mordvilko (1916)*			-	-	Unable to locate types.
= *Schizaphissetariae*	Rusanova (1962)*			-	-	Unable to locate types.
= *Aphisvulpiae*	del Guercio (1914b)			-	-	Unable to locate types.
= *R.zeae*	Rusanova (1960)*			-	-	Unable to locate types.
***R.musae***	Schouteden (1906)	NHMUK	NHMUK	27	7	Lecto- and paralectotypes.
= *R.scirpifolii*	Gillette and Palmer (1932)	USNM	NHMUK, USNM	6	15	
***R.nigrum***	Richards (1960)	CNC	CNC, NHMUK, USNM	10	8	
***R.nymphaeae***	Linnaeus (1761)			58	14	
= *Aphisaquatica*	Jackson (1908)	OSUC (?)		-	-	Unable to locate types. Type depository not given in original description. Jackson worked at The Ohio State and likely deposited his types there, however a search for the specimens did not locate them.
= *Aphisbutomi*	von Schrank (1801)			-	-	Unable to locate types.
= *Aphisinfuscata*	Koch (1854a)			-	-	Unable to locate types.
= *R.najadum*	Koch (1854b)			-	-	Unable to locate types.
= *R.momo*	Shinji (1922)			-	-	Unable to locate types, apparently lost.
= *Aphisprunaria*	Walker (1848)	NHMUK		0	2	
= *Aphisprunorum*	Dobrowljansky (1913)	Kiev Entomological Station (defunct)		-	-	Unable to locate types, apparently lost.
= *Hyadaphissparganii*	Theobald (1927)	NHMUK	NHMUK	6	0	
= *R.yoksumi*	Ghosh et al. (1971)	Entomology laboratory, Calcutta University		-	-	Types not examined.
***R.oxyacanthae***	von Schrank (1801)	NHMUK		0	9	
= *Aphiscrataegella*	Theobald (1912)	NHMUK		-	-	
= *Aphisedentula*	Buckton (1879)	NHMUK	NHMUK	-	-	Holotypes and 2 paratypes on one slide, holotype indicated. Ovipara.
= *Aphisfitchii*	Sanderson (1902)	USNM	NHMUK	0	3	
= *Aphisinsertum*	Walker (1849)	USNM		0	54	
= *Aphismacatata*	Walker (1849)	NHMUK		0	1	
= *R.viridis*	Richards (1960)	CNC	CNC, NHMUK	0	14	
= *R.malibivincta*	Fitch (1855)	USNM		-	-	Unable to locate types, apparently lost. See also Favret et al. (2008)
= *R.malifulviventris*	Fitch (1855)	USNM		-	-	
= *R.maliimmaculata*	Fitch (1855)	USNM		-	-	Unable to locate types, apparently lost. See also Favret et al. (2008)
= *R.malinigricollis*	Fitch (1855)	USNM		-	-	
= *R.malinigriventris*	Fitch (1855)	USNM		-	-	
= *R.maliobsoleta*	Fitch (1855)	USNM		0	1	
= *R.malipallidicornis*	Fitch (1855)	USNM		0	1	
= *R.malitergata*	Fitch (1855)	USNM		0	1	
= *R.malithoracica*	Fitch (1855)	USNM		0	1	
= *R.malitriseriata*	Fitch (1855)	USNM		0	1	
***R.padi***	Linnaeus (1758)	NHMUK		49	44	Holotype not examined.
= *Siphocoryneacericola*	Matsumura (1917)			-	-	Unable to locate types.
= *Siphonaphispadiamericana*	Mordvilko (1921)			-	-	Unable to locate types.
= *Aphisavenaestivae*	von Schrank (1801)			-	-	Unable to locate types.
= *Siphocorynedonarium*	Matsumura (1918)			-	-	Unable to locate types.
= *Siphocorynefraxinicola*	Matsumura 1917			-	-	Unable to locate types.
= *Aphisholci*	Ferrari (1872)			-	-	Unable to locate types.
= *Aphisprunifoliae*	Fitch (1855)	USNM		24	24	
= *Aphispseudoavenae*	Patch (1917)	CNC		2	11	
= *Aphistritici*	Lawson (1866)*			-	-	Unable to locate types.
= *Aphisuwamizuskurae*	Monzen (1929)			-	-	Unable to locate types.
***R.padiformis***	Richards (1962)	CNC	CNC, NHMUK, USNM	4	0	
***R.parvae***	Hottes and Frison (1931)	USNM		3	0	
***R.rufiabdominale***	Sasaki (1899)	USNM	USNM	24	28	
= *Cerosiphacalifornica*	Essig (1944)	EMEC	US Bureau of Entomology and Plant Quarantine (defunct)	0	1	Unable to locate paratypes, apparently lost.
= *R.fucanoi*	Moritsu (1947)*	Entomology Laboratory, Kyusyu Imperial University		-	-	Types not examined.
= *R.gnaphalii*	Tissot (1933b)	USNM		4	1	
= *Anuraphismume*	Hori (1927)			-	-	Unable to locate types.
= *Yamataphisoryzae*	Matsumura (1917)			-	-	Unable to locate types.
= *Yamataphispapaveri*	Takahashi (1921)*	Taihoku agricultural experiment station (defunct)		-	-	Unable to locate types.
= *Pseudocerosiphapruni*	Shinji (1932)*			-	-	Unable to locate types.
= *Areshasetigera*	Blanchard (1939)			-	-	Unable to locate types.
= *Areshashelkovnikovi*	Mordvilko (1921)			-	-	Unable to locate types.
= *Siphocorynesplendens*	Theobald (1915)	NHMUK		40	3	
= *R.subterraneum*	Mason (1937)	USNM		17	19	
***R.rufulum***	Richards (1960)	CNC	CNC, NHMUK	10	10	
***R.sanguinarium***	Baker (1934)		NHMUK (cotype)	0	1	
**sp. nov. "ex. Arisaema**"	undescribed	USNM		34	1	
**R. sp. T**	undescribed, proposed by Bulman et al. (2005)	Crop and Food Research, Lincoln, New Zealand		-	-	
**R. near insertum**	undescribed, proposed by Bulman et al. (2005)	Crop and Food Research, Lincoln, New Zealand		-	-	
**R. sp. x**	undescribed, proposed by Valenzuela et al. (2009)			-	-	

**Table 2.** *Rhopalosiphum* hosts and distributions. Host author names have been omitted for space. Unless otherwise noted, information is compiled from the original description (see Table 1), Holman (2009), and Blackman and Eastop (2017).

**Taxon**	**Primary host**	**Secondary host**	**Distribution**	**Notes**	**Additional references**
*R.albigubernum*	*Citrus*		Baidicheng, Fengjie, Sichuan, China	Known only from the type series, which consists of two alate vivipara.	
*R.arundinariae*		*Arundinariatecta*	Gainesville, FL	Known only from the type series, which were found as dense colonies on ventral side of younger *A.tecta* leaves that contained aptareae and alatae on 16 April 1930.	
*R.cerasifoliae*	*Prunus pennsylvanica, P. virginiana*	Cyperaceae, including *Schoenoplectus*, *Scirpus*, and *Eleocharis*. Also *Juncus* (Juncaceae)	North America, wherever host plants occur	Can persist throughout summer on primary host.	
*R.chusqueae*		*Chusqueatomentosa*	Costa Rica	Lives close to the nodes and well protected by leaves of *C.tomentosa*, so not easily detectable. Alatae and lifecycle unknown	
*R.dryopterae*	*Dryopterisfilix-mas*		Kyrgyzstan		
*R.enigmae*		*Typha*, especially. *T.latifoli*a; also recorded from *Sparganium*	North America, wherever host plants occur, though apparently most common in the East (A. Jensen, pers. comm.). "R.laconae" is morphology restricted to southeastern coastal plain.	Autoecious on cattails, no primary host known.	
*R.maidis*	*Prunus*, including *P.cornuta* (Pakistan), *P.mume* and *P.persica* (Korea), and *P.sargentii* (Japan).	*Zea*, *Sorghum*, *Hordeum*,other Poaceae; occasionally Cyperaceae and Typhaceae.	Cosmopolitan, but cannot survive outdoors in regions with severe winter climates	Most lineages are autoecious anholocyclic, with males and ovipara occurring only rarely. Heteroecious, androcyclic populations are known from Asia, where the species originated. In the Northern Hemisphere, lineages with different chromosome counts are associated with different hosts: 2n = 10 colonize *Hordeum* and *Echinochloacrus-galli*, while 2n = 8 colonize *Zea* and *Sorghum*; lineages with 2n = 9 , 2n = 11, and heterozygous 2n = 8 are also known. In contrast, 2n = 8 and 2n = 9 lineages in Australia do not exhibit host preference. Morphological and early genetic investigations of North American lineages reported incomplete morphological separation and lack of genetic differences between the various parthenogenetic karyotype lineages of *R.maidis*, though future investigations may raise one or more lineages to species from within the complex.	Wildermuth and Walter (1932), Blackman (1954), Menon and Ghai (1969), Brown and Blackman (1988), Blackman et al. (1990), Blackman and Brown (1991), Barro (1992), Simon et al. (1995), Jauset et al. (2000)
*R.musae*	*Prunus beseyi, P. subcordata, P. fasciculata*	*Scirpus*; also *Musa*, *Ensete*, *Sterlitzia*	WA to CO, MD (native); Europe, Middle East, Africa, and Australia (adventive)	Adventive populations outside of the native range are presumed to be anholocyclic. Specimen vouchers reportedly sent to the USNM are not present in the collection and were presumably never sent.	Taber (2003)
*R.nigrum*	*Crataegus*	*Avenasativa*, *Zizaniaaquatica*, *Alisma*	ON, MB, AK; unconfirmed reports from OR, UT		Pantoja et al. (2006)
*R.nymphaeae*	*Prunus*	Aquatic and semi-aquatic plants, including *Alisma*, *Juncus*, *Nelumbo*, *Nuphar*, *Sagittaria*, *Sparganium* and *Typha*. Occasionally other hosts, including *Canna*, *Glyceria*, *Lactuca*, *Triticum*, and *Tulipa*	Cosmopolitan		
*R.oxyacanthae*	Various Rosaceae including *Malus*, *Pyrus*, *Cotoneaster*, *Crataegus*, *Sorbus*, and *Prunus*	Various Poaceae, including *Agropyron*, *Agrostis*, *Festuca*, *Poa*; occasionally cyperaceae and Juncaceae.	North America (probable native range), Europe, North Africa, Japan	The name *R.insertum* Walker was used widely for the species in North America, but García Prieto et al. (2004) synonymized the species with *R.oxyacanthae*.	
*R.padi*	Primarily *Prunusvirginiana* (North America) and *P.padus* (Europe), occasionally other *Prunus*. One record on *Syringavulgaris*.	Primarily Poaceae, occasionally Asteraceae, Brassicaceae, Cyperaceae, Iridaceae, Juncaceae, Lilaceae, Typhaceae and other taxa.	Cosmopolitan	Can persist throughout summer on primary host.	
*R.padiformis*		*Poa*, *Triticum*	BC, MT	Primary host and associated morphs not known; alate males have been obtained in culture.	
*R.parvae*		*Carex* (US), *Scirpuslacustris* (Italy)	Native to North America (IL); Italy (adventive)	Primary host not known.	
*R.rufiabdominale*	Primarily *Prunus*; also recorded from *Malus*, *Chaenomeles*, *Pyrus*, *Rhodotypos*, and *Sorbus*.	Underground parts of Poaceae (including cereals) and Cyperaceae. Can infest some dicots (e.g. Asteraceae, Solanaceae), especially in greenhouse and hydroponic situations.	Native to East Asia. Currently pan-tropical/subtropical and restricted to greenhouses in colder climates.	Heteroecious holocyclic in East Asia and Italy; autoecious anholocyclic in majority of introduced range. Rakauskas et al. (2015) noted that if the heteroecious holocyclic forms recently reported from Italy spreads the species may persist in cold climates in Europe.	Etzel and Petitt (1992), Ciampolini et al. (1993), Zilahi-Balogh et al. (2005)
*R.rufulum*	*Crataegus*	Primarily *Acorus*, also recorded from *Typha*	North America and Europe, wherever hosts are found.		
*R.sanguinarium*	*Crataegusmexicana*	Unknown, but reared on various Poaceae in lab conditions	Mexico		
sp. nov. "ex. Arisaema"	Unknown	*Arisaema*	Maryland		
R. sp. T	Unknown	Cereals	New Zealand		
R. near insertum	Unknown	Cereals	New Zealand; Victoria, Australia		
R. sp. x	Unknown	*Zeamays*	Victoria, Australia		

**Table 3.** Descriptions of measurements.

**Measurement number**	**Measurement description or ratio**
1	Antenna segment (AS) 1, length
2	Antenna segment (AS) 2, length
3	Antenna segment (AS) 3, length
4	Antenna segment (AS) 4, length
5	Antenna segment (AS) 5, length
6	Antenna segment (AS) 6 base, length
7	Antenna segment (AS) 6, process terminalis (pt), length
8	Head width across eyes
9	Ultimate rostral segment (RIV+V), length
10	Ultimate rostral segment (RIV+V), width
11	Hind femur, length
12	Hind tibia, length
13	Hind distitarsus, length
14	Siphunculus, length
15	Cauda, length
16	Abdominal segment 8 submedian seta, length
17	Body length (BL)
18	Siphunculus, width
19	RIV+V length: RIV+V width
20	RIV+V length: hind distitarsus length
21	Pt length: AS 6 base length
22	Siphunculus length: siphunculus width
23	Siphunculus length: cauda length
24	Siphunculus length: AS 3 length
25	RIV+V length: cauda length
26	BL: head width
27	AS 3 and 4 fused (0 = no, 1 = yes)
28–33	Angle, A1–A6 (see Fig. 2b)
34–36	Area, S1–S3 (see Fig. 2c)
37–57	Wing length, L1–L21 (see Fig. 2d)
58	L1:L2 (37:38)
59	L1:L3 (37:39)
60	L1:L4 (37:40)
61	L1:L5 (37:41)
62	L1:L6 (37:42)
63	L1:L7 (37:43)
64	L2:L3 (38:39)
65	L2:L4 (38:40)
66	L2:L6 (38:41)
67	L2:L7 (38:42)
68	L3:L4 (39:40)
69	L6:L7 (42:43)

**Table 4.** Wing angle measurements for species of *Melanaphis*, *Rhopalosiphum*, and *Schizaphis*. Ranges are followed parenthetically by the average for each measurement. Generic measurements, which are listed in bold for easy comparisons, were calculated by adding together species in each genus. *Rhopalosiphumarundinariae* Tissot 1933a was not included in any generic summary and is presented by itself to allow easy comparisons to *Melanaphis* and *Rhopalosiphum*.

**Genus species**	**Original description**	**Specimens measured**	**A1**	**A2**	**A3**	**A4**	**A5**	**A6**
***Melanaphis***	**van der Goot (1917)**	**62**	**25.2–42.6 (30.6)**	**92.9–118.7 (106.9)**	**44.9–60.6 (52.6)**	**37.9–53.1 (43.5)**	**27–48.1 (36.8)**	**36–53.1 (42.7)**
*M.bambusae*	Fullaway (1910)	9	27–34.7 (31.6)	92.9–104 (101)	55.6–60.6 (57.9)	41.1–53.1 (45.4)	34.1–47.1 (41.3)	41–53.1 (45.7)
*M.donacis*	Passerini (1861)	4	27–30.8 (29.6)	108.7–113.9 (111.1)	44.9–49 (46.9)	42.6–45 (43.4)	27–30.2 (28.7)	36–44.7 (41.5)
*M.japonica*	Takahashi (1919)	2	32–32 (32)	111.9–111.9 (111.9)	54.6–56.7 (55.7)	42.6–45.2 (43.9)	30.1–31.7 (30.9)	40.6–43.3 (42)
*M.pyraria*	Passerini (1861)	10	27.8–35.1 (32.1)	106.9–118.7 (112.6)	46–53.8 (49.7)	42–47 (44.3)	27.9–38.3 (33.4)	38.9–48.6 (44.9)
*M.sacchari*	Zehntner (1897b)	36	25.2–42.6 (29.7)	94.8–112.1 (105.7)	45.6–56.3 (52.4)	37.9–48.2 (42.8)	29.9–48.1 (37.9)	37.4–49.7 (41.6)
*M.sorini*	Halbert et al. (2000)	1	29.5–29.5 (29.5)	113.7–113.7 (113.7)	48.2–48.2 (48.4)	42.8–42.8 (42.8)	33.4–33.4 (33.4)	42.2–42.2 (42.2)
***R.arundinariae***	**Tissot (1933a)**	**5**	**29.5–34 (31.5)**	**102.2–114.1 (108.5)**	**45.3–55.7 (48.8)**	**43.8–45.6 (44.5)**	**35.1–37.5 (36.5)**	**44.7–49.1 (47.1)**
***Rhopalosiphum***	Koch (1854a)	**342**	**26.6–44.6 (34.6)**	**96.7–123.5 (113.1)**	**28.8–44.6 (36.1)**	**38.7–55.1 (47.1)**	**15.2–47.4 (25.9)**	**37.3–62.8 (50.3)**
*R.cerasifoliae*	Fitch (1855)	23	29.7–40.2 (33.6)	106.3–122 (114.2)	33.4–41.2 (37.8)	41.8–48.8 (45.3)	17.7–42.1 (26.7)	43.7–52.6 (47.8)
*R.enigmae*	Hottes and Frison (1931)	38	26.6–38.3 (32.3)	110.3–123.5 (115.8)	33–43 (37.6)	39.9–51.8 (45.3)	19.9–47.4 (30.4)	37.3–53.7 (48.6)
*R.maidis*	Fitch (1856)	53	28.1–44.6 (34.1)	101.7–123.2 (112.7)	28.9–40.5 (35.2)	41.9–54.3 (46.3)	15.6–36.9 (24.9)	40.8–60.1 (49.7)
*R.musae*	Schouteden (1906)	15	30.6–39.2 (34.5)	111.2–119.6 (116)	31.1–36.3 (34.1)	42.6–50.8 (46)	15.2–25.3 (19.9)	43.4–53.4 (49.2)
*R.niger*	Richards (1960)	8	29.2–35.2 (31.7)	112.8–122.1 (116.4)	36.6–42 (38.5)	44.5–49.2 (46.9)	21.5–32.6 (26.8)	48.4–55.5 (51.2)
*R.nymphaeae*	Linnaeus (1761)	14	29.6–40.8 (34.8)	105.3–118 (112.4)	35.3–44.4 (40.3)	44.3–52 (47.6)	19.6–46.7 (25.7)	46.1–58 (49.1)
*R.oxyacanthae*	von Schrank (1801)	73	30.7–42.6 (35.9)	105.5–120.9 (112.6)	32.6–44.6 (36)	42.4–51.5 (47.5)	17–41.3 (25.6)	42.3–58.6 (50.6)
*R.padi*	Linnaeus (1758)	72	27–43.3 (35.7)	96.7–123.1 (110.9)	28.8–41.1 (35.1)	38.7–52.6 (48)	17.5–35.7 (23.6)	42.9–62.8 (51.7)
*R.rufiabdominale*	Sasaki (1899)	43	28.5–39.7 (34.4)	105.5–120.3 (113.8)	29.6–40.1 (35)	43.7–55.1 (48.9)	18.6–36.1 (27.6)	45.6–58.5 (51.6)
*R.sanguinarium*	Baker (1934)	1	40.9–40.9 (40.9)	101.3–101.3 (101.3)	39.8–39.8 (39.8)	44.6–44.6 (44.6)	29.6–29.6 (29.6)	47.8–47.8 (47.8)
***Schizaphis***	Börner (1931)	**99**	**26.3–41.5 (31.7)**	**100–123.4 (112.4)**	**29.5–44.9 (38.8)**	**38.1–52.2 (44.2)**	**18.5–53.8 (37.9)**	**33.6–58 (46.3)**
*S.caricis*	Schouteden (1906)	2	28.6–33.8 (31.2)	111.1–117.9 (114.5)	37–40.2 (38.6)	46.3–46.3 (46.3)	29.4–29.4 (29.4)	51.4–51.4 (51.4)
*S.graminum*	Rondani (1852)	41	28.4–35.5 (31.1)	103.7–118.5 (111.9)	36.3–44.9 (40.6)	38.1–44.6 (41.4)	32.7–53.8 (39)	40.8–51.1 (44)
*S.minuta*	van der Goot (1917)	1	31.5–31.5 (31.5)	115–115 (115)	41.7–41.7 (41.7)	42.3–42.3 (42.3)	38.9–38.9 (28.9)	45.8–45.8 (45.8)
*S.muhlenbergiae*	Phillips and Davis (1912)	2	27.3–27.3 (27.3)	109.6–109.6 (109.6)	35.7–43.4 (39.6)	38.25–40.9 (39.6)	41.1–44.6 (42.9)	43.7–49.3 (46.5)
*S.nigra*	Baker (1918)	44	26.3–41.5 (31.7)	100–123.4 (113.5)	29.5–42.1 (37.5)	43–52.2 (46.5)	18.5–46.8 (37.5)	33.6–58 (47.9)
*S.palustris*	Theobald (1929)	3	30–38.5 (34.1)	105.3–112.1 (107.7)	33.7–39.5 (37.1)	46.5–49.2 (48.3)	27.8–43.8 (35)	46.8–53 (49.5)
*S.rotundiventris*	Signoret (1860)	6	30.6–40.4 (35)	107–115.8 (110.8)	33.6–37.7 (36.3)	44.5–50.6 (47.2)	31.9–38.9 (35.5)	46.2–53.6 (48.7)

**Table 5.** Morphological differences between *R.parvae* and *R.rufulum*. Measurement and ratio ranges are followed parenthetically by the mean and number of specimens measured.

**Species**	**ant-6**	**caud-l**	**bl**	**rost-l:rost-w**	**ant-pt:ant-6**	**bl:head**	**bl:ant-pt**
*R.parvae*	61.7–66.8(64.7, 3)	91.8–103.1(98.4, 3)	1410.2–1437.0(1427.9, 3)	2.13–2.16(2.15, 2)	3.37–3.77(3.56, 3)	3.48–3.84(3.60, 3)	5.80–6.90(6.22, 3)
*R.rufulum*	77.1–91.0(83.6, 9)	94.9–131.6(119.7, 9)	1829.0–2186.0(1969.3, 10)	1.37–2.06(1.78, 9)	2.42–3.45(3.14, 9)	4.15–5.04(4.61, 10)	6.65–9.71(7.50, 9)
